# Bovine Epithelial *in vitro* Infection Models for *Mycoplasma bovis*

**DOI:** 10.3389/fcimb.2018.00329

**Published:** 2018-09-18

**Authors:** Christoph Josi, Sibylle Bürki, Ana Stojiljkovic, Olga Wellnitz, Michael H. Stoffel, Paola Pilo

**Affiliations:** ^1^Department of Infectious Diseases and Pathobiology, Vetsuisse Faculty, Institute of Veterinary Bacteriology University of Bern, Bern, Switzerland; ^2^Graduate School for Cellular and Biomedical Sciences, University of Bern, Bern, Switzerland; ^3^Division of Veterinary Anatomy, Department of Clinical Research and Veterinary Public Health, Vetsuisse Faculty, University of Bern, Bern, Switzerland; ^4^Veterinary Physiology, Department of Clinical Research and Veterinary Public Health, Vetsuisse Faculty, University of Bern, Bern, Switzerland

**Keywords:** *Mycoplasma bovis*, genotype, epithelial cells, mammary cells, tissue predilection, pathogenicity

## Abstract

*Mycoplasma bovis* causes bovine mycoplasmosis. The major clinical manifestations are pneumonia and mastitis. Recently an increase in the severity of mastitis cases was reported in Switzerland. At the molecular level, there is limited understanding of the mechanisms of pathogenicity of *M. bovis*. Host–pathogen interactions were primarily studied using primary bovine blood cells. Therefore, little is known about the impact of *M. bovis* on other cell types present in infected tissues. Clear *in vitro* phenotypes linked to the virulence of *M. bovis* strains or tissue predilection of specific *M. bovis* strains have not yet been described. We adapted bovine *in vitro* systems to investigate infection of epithelial cells with *M. bovis* using a cell line (MDBK: Madin-Darby bovine kidney cells) and two primary cells (PECT: bovine embryonic turbinate cells and bMec: bovine mammary gland epithelial cells). Two strains isolated before and after the emergence of severe mastitis cases were selected. Strain JF4278 isolated from a cow with mastitis and pneumonia in 2008 and strain L22/93 isolated in 1993 were used to assess the virulence of *M. bovis* genotypes toward epithelial cells with particular emphasis on mammary gland cells. Our findings indicate that *M. bovis* is able to adhere to and invade different epithelial cell types. Higher titers of JF4278 than L22/93 were observed in co-cultures with cells. The differences in titers reached between the two strains was more prominent for bMec cells than for MDBK and PECT cells. Moreover, *M. bovis* strain L22/93 induced apoptosis in MDBK cells and cytotoxicity in PECT cells but not in bMec cells. Dose-dependent variations in proliferation of primary epithelial cells were observed after *M. bovis* infection. Nevertheless, an indisputable phenotype that could be related to the increased virulence toward mammary gland cells is not obvious.

## Introduction

*Mycoplasma bovis* was first isolated in 1961 in the United States from a dairy herd with an outbreak of mastitis (Hale et al., 1962). *M. bovis* is one of the major causative agents of bovine mycoplasmosis. Clinical manifestations are broad, including bronchopneumonia, mastitis, otitis, arthritis, keratoconjunctivitis, meningitis, and genital disorders (Bürki et al., 2015a). This bacterium is an emerging pathogen in industrialized countries, leading to high economic losses in dairy and beef cattle production. Management of bovine mycoplasmosis is challenging as chronic infections in combination with subclinical development of the disease are often observed (Maunsell et al., 2011; Nicholas, 2011). Furthermore, current vaccines are ineffective in the field and antibiotic treatments generally fail, while resistance to antimicrobials is increasing (Gautier-Bouchardon et al., 2014; Perez-Casal et al., 2017).

In Switzerland, *M. bovis* was predominantly associated with pneumonia and subclinical mastitis (Burnens et al., 1999). In the mid-2000s, a rise in the severity of mastitis cases due to *M. bovis* was observed (Aebi et al., 2012, 2015). A similar trend was documented in Northern Italy (Radaelli et al., 2011), Austria (Spergser et al., 2013), and Israel (Lysnyansky et al., 2016). Molecular epidemiology studies of Austrian and Swiss strains revealed distinct genotypes suggesting a switch in the circulating *M. bovis* genotypes in Switzerland in parallel with an increased number of severe mastitis cases (Bürki et al., 2016). However, it remains unclear whether the currently circulating *M. bovis* strains show higher predilection or virulence toward mammary gland cells than older strains (Bürki et al., 2016). Tissue predilection of specific *M. bovis* strains has not been previously reported. Past research focused mainly on blood cells and partially neglected a potential role of other cell types like epithelial cells in disease development.

To establish an efficient infection, bacteria have to adhere to host cells, multiply or persist in the host, and evade the host immune system. Several mechanisms of pathogenicity of *M. bovis* have been described and disease development seems to be multifactorial (Bürki et al., 2015a). Adhesion is one of the first steps of mycoplasma infection (Rottem, 2003). Several surface exposed proteins were characterized as adhesins (Sachse et al., 1993, 1996, 2000; Thomas et al., 2003b). However, the molecular mechanisms of cell-dependent adhesion are still not understood due to a lack of knowledge of the corresponding eukaryotic receptors. Recently, three adhesins were identified: α-enolase, NOX and TrmFO. They were shown to bind to plasminogen and fibronectin, serving as a bridge between the bacterial adhesins and the host cell receptors (Song et al., 2012; Guo et al., 2017; Zhao et al., 2017). Binding to plasminogen and fibronectin might facilitate invasion and dissemination in the host, as described for other bacteria (Raymond and Djordjevic, 2015). Occasional intracellular localization of *M. bovis* in inflammatory host cells was previously shown *in vivo* (Adegboye et al., 1995; Rodríguez et al., 1996; Maeda et al., 2003; Kleinschmidt et al., 2013). More recently, *in vitro* uptake of *M. bovis* by several bovine blood cell types was demonstrated (van der Merwe et al., 2010; Suleman et al., 2016; Jimbo et al., 2017; Bürgi et al., 2018). Moreover, invasion of primary embryonic calf turbinate (PECT) cells, the Embryonic Bovine Lung (EBL) cell line, and the Embryonic Bovine Tracheal (EBTr) cell line was shown (Bürki et al., 2015b; Suleman et al., 2016). To date, the molecular mechanisms involved in *M. bovis* invasion of bovine cells and a potential differential permissivity dependent on the cell type have not been described. Invasion of *M. bovis* in different cell types might contribute to the pathogenicity of the bacterium. *M. bovis* may reside in a protective niche evading the host immune response and antimicrobial treatment. Furthermore, invasion of host blood cells could also lead to systemic spread of this bacterium (Bürki et al., 2015a).

*In vitro* studies indicated that cytotoxicity, apoptosis and host cell proliferation after infection with *M. bovis* differ among host cell types (Bürki et al., 2015a). Infected PBMCs were studied, considering cytotoxic effects, induction of apoptosis and viability of host cells, but results were inconsistent (Vanden Bush and Rosenbusch, 2002; van der Merwe et al., 2010; Gondaira et al., 2015). However, *M. bovis* was shown to inhibit proliferation of PBMCs (Vanden Bush and Rosenbusch, 2002; van der Merwe et al., 2010; Suleman et al., 2016, 2018). Studies analyzing distinct blood cell types gave clearer results. *M. bovis* infection delayed apoptosis in monocytes (Mulongo et al., 2014) and alveolar macrophages (Suleman et al., 2016), whereas a reduction in host cell viability and induction of apoptosis was observed in infected neutrophils (Jimbo et al., 2017). Additionally, a slight induction of apoptosis and cytotoxicity after *M. bovis* infection was observed in bovine macrophage cell lines (Bürgi et al., 2018). Compared to blood cell types, little is known about the effects of *M. bovis* on epithelial cells. *M. bovis* infection induced a slight cytotoxic effect in bovine endothelial cells, whereas a strong increase in apoptosis was observed in human epithelial cells (Sokolova et al., 1998; Lu and Rosenbusch, 2004).

Knowledge of *M. bovis* effects on epithelial cells is fragmentary. In the present study, host–*M. bovis* interactions using bovine epithelial cells from different organs were investigated. The aim of the study was to identify virulence phenotypes in bovine epithelial cells resulting from infection with two strains of *M. bovis*, with a focus on bovine epithelial mammary gland cells.

## Materials and methods

### Bacterial strains and axenic growth conditions

*M. bovis* strains L22/93 and JF4278 were used. Strain L22/93 was isolated in 1993 in Switzerland from the lung of a cow. This strain was assigned to the sequence type (ST) 17 by multilocus sequence typing (MLST) (Bürki et al., 2016). Strain JF4278 was isolated from the milk of a cow with mastitis and pneumonia in Switzerland in 2008 (Aebi et al., 2012). This strain was originally submitted to the database as ST 5 on the basis of sequencing PCR amplicons (Bürki et al., 2016). It was later discovered that the *adh-1* allele assignment was incorrect and that the isolate actually lacks that locus. The current, updated pubMLST profile (https://pubmlst.org/bigsdb?db=pubmlst_mbovis_isolates), including the absence of the adh-1 locus, is consistent with the genome sequence data (Genbank accession number: NZ_LT578453.1). This finding was subsequently confirmed by repeating the analysis of the isolate based on PCR. The missing *adh-1* locus means that the isolate is not typeable by the MLST reference method. Other isolates similarly lacking the *adh-1* locus have also been identified. As a result, an effort to modify the scheme to restore the ability to type all isolates is underway. There are a number of other isolates in the pubMLST database for which no adh-1 locus is given. Strains of *M. bovis* were grown at 37°C in SP4 medium (Freundt, 1983) supplemented with 50 μg/mL cefoxitin sodium salt (Sigma-Aldrich, Buchs, Switzerland). Pre-cultures were grown for 20 h from frozen stocks diluted 1:100 in SP4 broth medium. The concentration of all pre-cultures was measured by performing 10-fold serial dilutions and plating on SP4 agar plates. SP4 agar plates were incubated at 37°C for 4–5 days in a humidified atmosphere. Colonies were counted under a stereomicroscope. For standardization purposes, growth of each frozen bacterial stock and SP4 batches were tested. The generation time of both strains was assessed in a previous study, JF4278 had a generation time of 1.52 h ± 0.08, while L22/93 had a generation time of 2.01 h ± 0.16 (Bürgi et al., 2018).

### Epithelial cells and cell infections

Three different epithelial cell types were used in this study. No ethics approval was needed because primary cells were collected from organs of bovine carcasses at the slaughterhouse in accordance with the Swiss Federal Animal Protection Law, RS455. The Madin-Darby Bovine Kidney (MDBK) epithelial cell line was derived from the kidney of an adult steer in 1957 (Madin and Darby, 1958). PECT cells were prepared from bovine fetuses from a local abattoir (Schweizer and Peterhans, 1999; Bürki et al., 2015b). Primary cultures of bovine mammary gland epithelial cells (bMec) were prepared from mammary gland tissues of cows directly after slaughter (Wellnitz and Kerr, 2004; Zbinden et al., 2015). MDBK and PECT cells were grown in minimal essential medium (MEM)-Earle medium supplemented with 2.2 g/L NaHCO_3_ (Biochrom, Berlin, Germany) and 7% fetal bovine serum (Thermo Fisher Scientific, Reinach, Switzerland), 1X penicillin-streptomycin (Thermo Fisher Scientific) and 1% L-glutamine (Biochrom). bMec cells were grown in Dulbecco's Modified Eagle's Medium/Nutrient Mixture F-12 Ham (DMEM/F-12) containing L-glutamine, HEPES and sodium bicarbonate (Sigma-Aldrich). DMEM/F-12 was supplemented with 10% fetal bovine serum, 1X penicillin-streptomycin and 1X insulin-transferrin-selenium (Thermo Fisher Scientific). All cells were maintained at 37°C in a humidified 5% CO_2_ atmosphere. Cells were routinely screened by PCR to ensure absence of mycoplasma contamination with the Venor®GeM kit (Minerva Biolabs, Berlin, Germany). All bovine cells used in this study were negative for *Mycoplasma* sp. The presence of bovine viral diarrhea virus (BVDV) in cell cultures was assessed using immunostaining with an in-house swine anti-BVDV hyperimmune serum (National Reference Center for BVDV, Institute of Virology and Immunology) as previously described (Bürki et al., 2015b; Bürgi et al., 2018). bMec cells were contaminated with BVDV but not PECT and MDBK cells. Cell passages 5–8 (PECT), 3–5 (bMec), and 117–127 (MDBK) were used for cell infections. Bovine cells were routinely seeded 24 h before the experiments (Supplementary Table 1). Before infection with *M. bovis*, the medium was changed to MEM-Earle supplemented with 7% fetal bovine serum and 1% L-glutamine without antibiotics, unless otherwise described. Cells from one well were counted before infection. 1.5 mL of *M. bovis* pre-cultures were centrifuged for 15 min at 5,900 × g and washed once in PBS (Thermo Fisher Scientific) at pH 7.5 unless otherwise described. Mycoplasmas were further suspended and diluted in MEM-Earle medium to infect cells at a multiplicity of infection (MOI) ranging between 1.3 and 9 (Supplementary Table 1). The MOI is defined as the number of added bacteria per individual host cell. The MOI was confirmed after growth on agar plates.

### Adhesion assays

Adhesion assays were adapted from a previous protocol (Sachse et al., 1996; Sachse, 1998). The quantification of adherent *M. bovis* was carried out using real-time qPCR. Briefly, eukaryotic cells were seeded as described above to reach high cell confluency (Supplementary Table 1). Mycoplasma pre-cultures were washed once and diluted in buffer A (0.05 M Tris-HCl, pH 7.2, 0.1 M NaCl, and 1 mM CaCl_2_). Before infection, cells were washed once with buffer A and blocked for 15 min with buffer A containing 0.1% bovine serum albumin (Sigma Aldrich). After an additional wash with buffer A, approximately 4 × 10^5^ mycoplasmas were added to the cells. *M. bovis* was allowed to adhere to bovine cells for either 30 min or 2 h on a rocker with 22 strokes/minute and an amplitude of 3.5 cm at 37°C. Cells were washed three times with buffer A to remove unattached mycoplasmas. Two hundred and fifty microliters of lysis solution [buffer A with 1% Tween® 20 (Sigma Aldrich) and 0.24 mg/mL Proteinase K (Roche Diagnostics, Rotkreuz, Switzerland)] were added. Plates were covered with a plastic seal and incubated for 80 min at 65°C with horizontal shaking at 500 rpm, followed by 15 min heat-inactivation at 96°C. These output samples were collected to quantify the amount of adherent mycoplasmas. The same amount of bacteria used for infection was directly lysed in a volume of 250 μL lysis solution (input samples). To quantify input and output samples, real-time qPCR was performed on a 7500 real-time PCR system (Thermo Fisher Scientific) as previously described (Rossetti et al., 2010). The data were analyzed with the 7500 System Software version v2.0.6 using auto settings for baseline values and the value of cycle threshold set at 0.157. Additionally, the specificity of the *M. bovis* qPCR was tested with uninfected cells (Supplementary Table 2). The percentage of adherent *M. bovis* relative to the input amount of *M. bovis* was calculated as follows (adapted from Nicholson et al., 2012):

(1) $$\begin{array}{r} \%\;of\;adherent\;M.\;bovis=2^{\left(a-b\right)}*100 \end{array}$$

where, *a* = *C*_t_ of input samples; and *b* = *C*_*t*_ of output samples. The assays were performed in triplicates in three independent experiments.

### Cell infection and gentamicin protection assay

Cell infections and gentamicin protection assays were adapted from previous studies using PECT and Bomac cells (Bürki et al., 2015b; Bürgi et al., 2018). The minimal inhibitory concentration (MIC) of gentamicin sulfate (TOKU-E Company, Bellington, USA) in SP4 was assessed by broth microdilution assay according to the Clinical and Laboratory Standards Institute (CLSI) guidelines (CLSI, 2011). After 48 h of incubation, the MIC for gentamicin was 8 and 4 μg/mL for strains JF4278 and L22/93, respectively. On the other hand, different concentrations of gentamicin sulfate were tested for efficient killing of the two strains within 3 h (Supplementary Figure 4). In line with the previous studies, 400 μg/mL gentamicin was required to efficiently kill the inoculum of *M. bovis* used for experiments within 3 h (van der Merwe et al., 2010; Bürki et al., 2015b). Mycoplasma pre-cultures and eukaryotic cells were prepared as described above, and the infection details are described in Supplementary Table 1. After infection, 24-well plates were centrifuged for 5 min at 600 × g to synchronize infection. Three hours post-infection, cells were washed twice with PBS. For cell infection assays, fresh MEM-Earle medium was added. For gentamicin protection assays, MEM-Earle medium supplemented with 400 μg/mL gentamicin sulfate was used to kill extracellular bacteria. In both assays, cells were incubated for 3 h (total 6 h post-infection). Subsequently, all samples were washed three times with PBS and fresh MEM-Earle medium was added to each well. Sampling was performed at time points 0, 6, and 54 h post-infection. Before sampling, wells were washed once with PBS at time points 0 and 54 h and three times for time point 6 h. Bovine cells were scraped and lysed mechanically using a 23-gauge needle and a syringe. Colony forming units (CFUs) per well were determined by performing 10-fold serial dilutions in PBS and subsequent plating on SP4 agar plates. As controls, assays were performed with *M. bovis* strains without bovine cells. These controls were used to check survival of the two strains in MEM-Earle medium and to validate the efficient killing of extracellular *M. bovis* by gentamicin. For this reason, the controls were performed for each experiment and each cell type. Since the controls were done in Eppendorf tubes, bacteria were centrifuged for 15 min at 5,900 × g before washing. The assays were performed in triplicates in three independent experiments.

### Confocal microscopy

To determine the localization of *M. bovis* after cell infection, an immunofluorescence staining protocol was established. Before seeding bovine cells, round glass slides of 12 mm diameter and #1.5 thickness (Neuvitro, Vancouver, USA) were placed into wells of 24-well plates. Seeding of cells was adjusted (Supplementary Table 1) to have isolated cells 54 h post-infection. As bMec cells showed reduced adherence to glass slides, more cells were seeded compared to other epithelial cells (Supplementary Table 1). Cell infection and gentamicin protection assays were performed as described above. At time point 54 h, infected cells were washed twice with PBS supplemented with 1 mM CaCl_2_ and 1 mM MgCl_2_. Cells were fixed for 10 min at room temperature using a 4% formaldehyde solution (Merck, Schaffhausen, Switzerland) and subsequently washed four times with PBS. Half of the samples were permeabilized, to allow intracellular staining. For permeabilization, 0.2% Triton® X-100 (Serva, Heidelberg, Germany) was added to the blocking buffer [5% inactivated horse serum (Thermo Fisher Scientific) in PBS]. Cells were incubated for 20 min at room temperature (Bürki et al., 2015b) and then washed four times with PBS. Glass slides were taken out of the wells and moved to a humidified plastic box for staining. First, all slides were blocked for 15 min using the blocking solution. Slides were incubated with a stock of in-house rabbit antiserum directed against PG45^T^ produced in 1975 (1:1,000 in blocking buffer) for 1 h. After each staining step, slides were washed four times with PBS. Then Alexa Fluor® 488-conjugated AffiniPure Goat anti-rabbit IgGs (H+L) (Jackson ImmunoResearch, Suffolk, UK) were added as secondary antibodies for 30 min (1:400 in blocking buffer). Cells were incubated with 1 mg/ml DAPI solution (Sigma-Aldrich) (1:1,000 in blocking buffer) for 15 min and with rhodamine phalloidin (PHDR1) (LuBio Science, Zürich, Switzerland) (100 nM in blocking buffer) for 30 min, to stain cell nuclei and F-actin, respectively. Finally, round glass slides were mounted on micro slides (Karl Hecht “Assistant” GmbH, Altnau, Switzerland) using Glycergel mounting medium (Agilent Technologies AG, Basel, Switzerland). Mounted slides were examined using an Olympus FV 1000 confocal laser scanning microscope (Olympus, Hamburg, Germany) with a 60X PlanAPO N objective with oil immersion using the 405, 488, and 555 nm laser channels. The softwares Fluoview-ASW 3.1 and ImageJ v1.51n were used to acquire and merge the fluorescence images.

### ApoTox-Glo™ triplex assay

The ApoTox-Glo™ Triplex Assay (Promega, Madison, USA) was used to assess viability, cytotoxicity and induction of apoptosis in bovine cells after infection with *M. bovis*. Viability and cytotoxicity were measured by fluorescent signals detectable after cleavage of protease substrates. To measure cell viability, the cell permeant substrate, is cleaved by a host protease only active in intact cells. The substrate used to measure cytotoxicity does not pass intact membranes and is cleaved when a protease is released into the medium. Induction of apoptosis is quantified by a luminescent signal derived from the cleavage of a substrate of activated caspase-3/7. Bovine cells were seeded 24 h before the start of the experiment in black 96-well plates with a clear bottom (Greiner Bio-One, Frickenhausen, Germany) as described before (Supplementary Table 1). After 24 h the cell medium was replaced with fresh medium and bovine cells were infected with *M. bovis* for an additional 24 h. To induce apoptosis in selected samples, staurosporine was added to the cells during the last 6 h of infection. Primary bovine cells (PECT and bMec) were treated with 10 μM staurosporine and MDBK cells with 2.5 μM staurosporine. The ApoTox-Glo™ Triplex assay was performed according to the manufacturer's protocol. Fluorescence and luminescence signals were read with a Cytation 5 Cell Imaging Multi-Mode Reader (BioTek Instruments GmbH, Luzern, Switzerland) with the filter settings suggested in the ApoTox-Glo™ Triplex Assay protocol. All the experiments were performed three times in duplicates.

### Proliferation assay

The iClick™ EdU Andy Fluor™ 647 Imaging Kit (GeneCopoeia™, Rockville, USA) was used to determine the effect of *M. bovis* on proliferation of bovine epithelial cells. EdU is an analog of thymidine that is incorporated into DNA during active DNA synthesis. Due to a copper-catalyzed click reaction, the Andy Fluor 647 dye can be covalently linked to the incorporated EdU. Thus, proliferating cells are stained with Andy Fluor 647. Bovine cells were seeded 24 h before the start of the experiment in black 96-well plates with a clear bottom (Supplementary Table 1). After 24 h, the cell medium was replaced with MEM-Earle without fetal bovine serum and incubated for 30 min. Before infection, the cell medium was replaced with MEM-Earle containing 2% fetal bovine serum, instead of 7% as used in other assays. Compared to the other assays, the MOI was reduced to ensure lower bacterial load per cell for subsequent image acquisition (Supplementary Table 1). Ten μM EdU were added to the cells during the last 4 h of infection. After a total of 24 h of infection, cells were washed once with buffer A (as used in adhesion assays). Cells were then fixed with 3.7% formaldehyde solution for 15 min and permeabilized with ice-cold 100% methanol for 10 min at −20°C. Before staining, cells were washed twice with buffer A containing 3% bovine serum albumin. Subsequently, the copper-catalyzed click reaction with the Andy Fluor 647 dye was performed according to the manufacturer's protocol in a total volume of 50 μL/well. Finally, staining of *M. bovis* and eukaryotic nuclei was performed as described above. However, dilutions of antibodies and dyes and washings between staining steps were done with buffer A containing 3% bovine serum albumin.

Cells were visualized using the INCell Analyzer 2000 system (General Electric Healthcare, Glattbrugg, Switzerland). Images were obtained using a wide field epifluorescence microscope with a Nikon objective lens (20X/NA 0.45), at a working distance of 7.5 mm. The following filter sets were used: FITC (490_20× & 525_36m) for the visualization of Alexa Fluor® 488 stained *M. bovis*, Cy5 (645_30× & 705_72m) for Andy Fluor 647 stained DNA and DAPI (350_50× & 455_60m) for Hoechst stained nuclei. Bright-field images were acquired to visualize the morphology of cells. Images were analyzed using the INCell Investigator 1.6.2 software (GE Healthcare). An example for image analysis is shown in Supplementary Figure 1. After image segmentation of the DAPI signal, a pseudo-cell was defined around the nuclei by expanding the nuclear mask by 44.4 μm in diameter. To ensure that adjacent cells were separated, a clump-breaking algorithm was applied. Within the area of each of the defined pseudo-cells, the intensity of the Cy5 and FITC signal was measured. In an additional step, Cy5 positive cells were checked for overlapping DAPI and Cy5 signals. The data of each cell was exported to a MS Excel file. Cells were grouped in Cy5 positive (proliferating) and negative (non-proliferating) cells. Additionally, the intensity of fluorescent signal for *M. bovis* within these cells was estimated. Thus, the amount of bacteria associated with each cell was measured. Finally, cells were grouped in quartiles according to their *M. bovis* association. All the experiments were performed three times in duplicates.

### Statistical analysis

For all assays, absolute or relative values are shown as means ± standard deviations of mean values from three independent experiments. The significance of differences between uninfected and infected cells with either strain was calculated with the Welch's *t*-test. Where indicated, the significance of differences between different cell types infected with the same strain or treated with the same compound was calculated with the Welch's *t*-test. Statistical analysis was performed using the software GraphPad Instat™ V2.05 (GraphPad Software Inc., La Jolla, CA, USA).

## Results

### Adhesion capacity of *M. bovis* is dependent on time and bovine cell type

**Figure 1:**
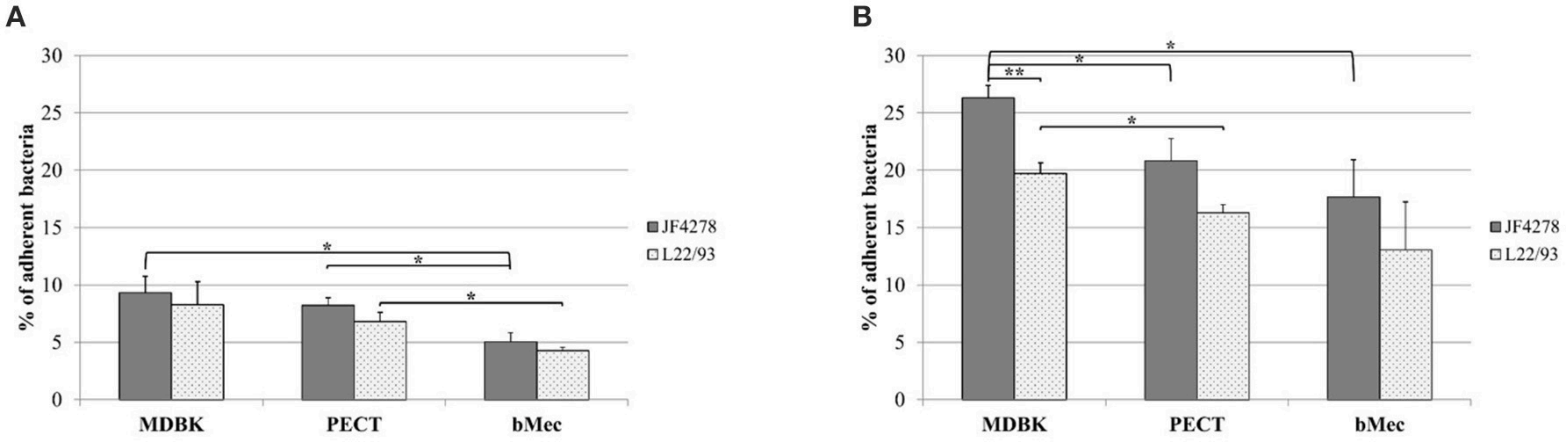
Adhesion assay. *M. bovis* adhesion to bovine epithelial cells after 30 min **(A)** and 2 h **(B)**. Gray columns correspond to strain JF4278, while spotted columns correspond to strain L22/93. The x-axis indicates the different bovine cell types used. The y-axis represents the percentage of adherent *M. bovis* relative to the added *M. bovis*. The data shown are the mean values of triplicates from three independent experiments. Standard deviations of measurements are indicated as vertical bars. *P < 0.05, **P < 0.01.

Adhesion of *M. bovis* strains JF4278 and L22/93 to bovine epithelial cell types was evaluated. As shown in Figure 1, adherence of *M. bovis* was expressed as the percentage of the initial inoculate of *M. bovis*. After 30 min, between 4.3% (strain L22/93 with bMec cells) and 9.3% (strain JF4278 with MDBK cells) of *M. bovis* adhered to bovine epithelial cells (Figure 1A). Adhesion capacity of *M. bovis* strain JF4278 to bMec cells was found to be significantly lower compared to the other cell types (Figure 1A). L22/93 showed a significantly lower adhesion capacity to bMec cells in comparison to PECT cells (Figure 1A). After 2 h of incubation, adhesion of *M. bovis* increased with percentages ranging between 13.1% (strain L22/93 with bMec cells) and 26.3% (strain JF4278 with MDBK cells) (Figure 1B). A higher adhesion capacity of *M. bovis*, especially for strain JF4278, to the bovine cell line compared to primary cells was observed (Figure 1B). Generally, adhesion of JF4278 was slightly higher than strain L22/93 (Figures 1A,B). However, this difference was shown to be significant only for MDBK cells after 2 h of incubation (Figure 1B).

### *M. bovis* is able to invade and grow in co-culture with different bovine epithelial cell types

**Figure 2:**
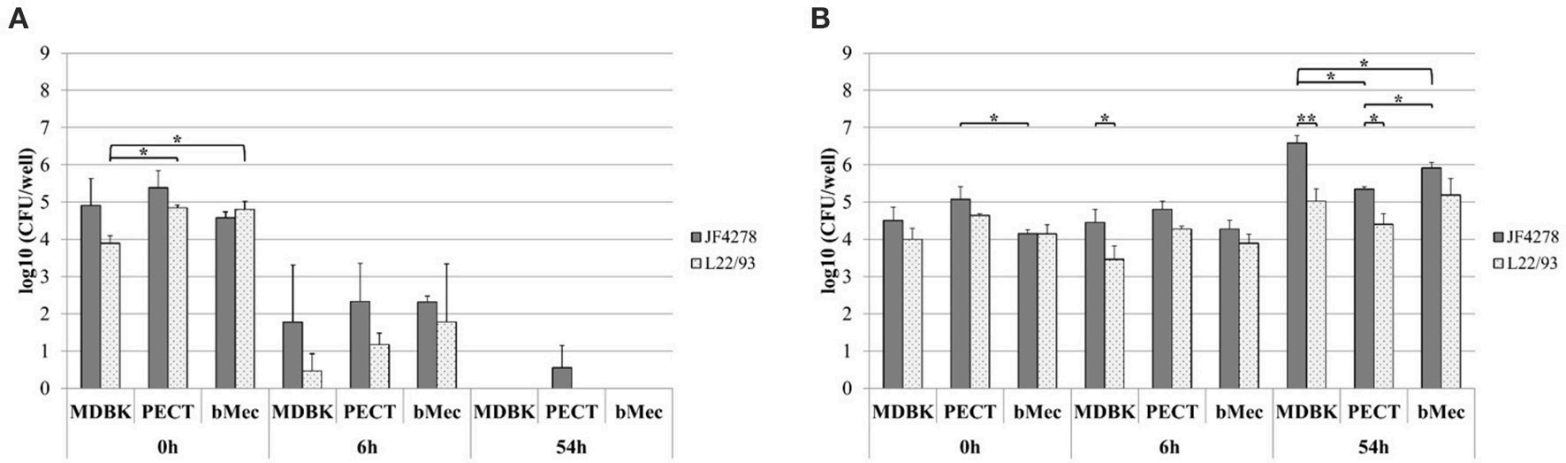
Infection model of *M. bovis* with bovine epithelial cell types. Survival of *M. bovis* in MEM-Earle medium without cells **(A)**. Survival and growth of *M. bovis* in co-culture with cells **(B)**. Gray columns correspond to strain JF4278, while spotted columns correspond to strain L22/93. The x-axis indicates the different bovine cell types and time points. The y-axis represents the log10 CFU/well of *M. bovis*. The data shown are the mean values of triplicates from three independent experiments. Standard deviations of measurements are indicated as vertical bars. Statistical analysis within the individual time points are shown. Bacterial concentrations of the two strains within each cell type were analyzed. Bacterial concentrations of the same strain between different cell types were analyzed. *P < 0.05, **P < 0.01.

**Figure 3:**
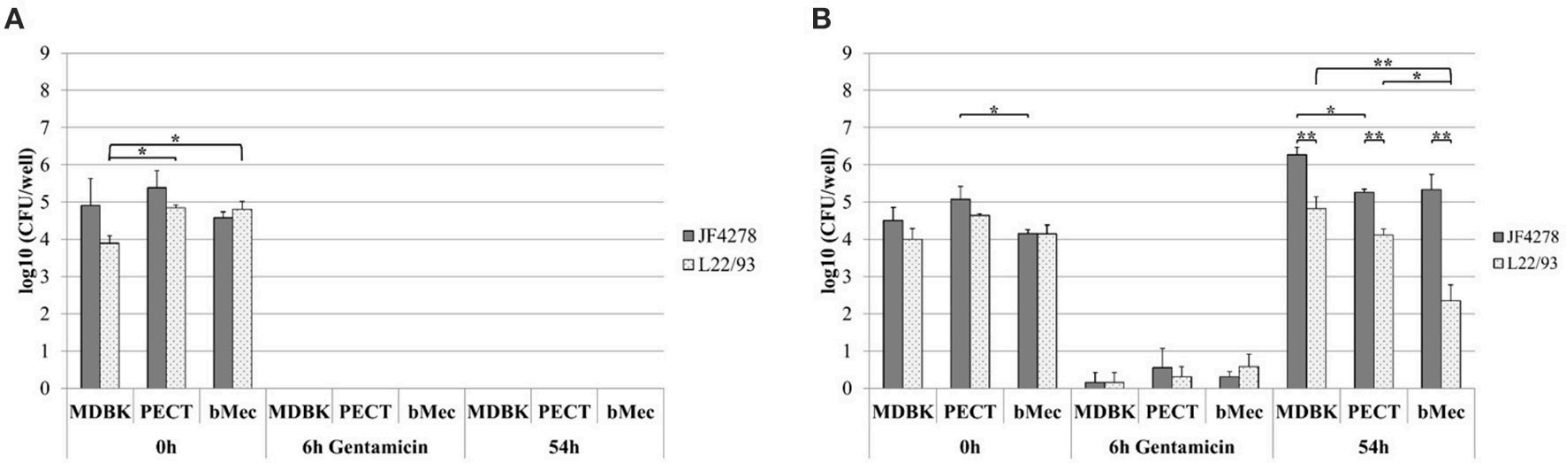
Gentamicin protection assay. Survival of *M. bovis* in MEM-Earle medium with gentamicin treatment without cells **(A)** and gentamicin protection assay **(B)**. Gray columns correspond to strain JF4278, while spotted columns correspond to strain L22/93. The x-axis indicates the different bovine cell types used and time points. The y-axis represents the log10 CFU/well of *M. bovis*. The data shown are the mean values of triplicates from three independent experiments. Standard deviations of measurements are indicated as vertical bars. Statistical analysis within the individual time points are shown. Bacterial concentrations of the two strains within each cell type were analyzed. Bacterial concentrations of the same strain between different cell types were analyzed. *P < 0.05, **P < 0.01.

*M. bovis* invasion and growth in co-culture were assessed. For each individual cell infection experiment and gentamicin protection assay, controls with *M. bovis* in cell culture medium without cells and with and without gentamicin were included (Figure 2A and Figure 3A). Since these controls were performed for each individual cell infection experiment, cell types are indicated in the graph although no eukaryotic cells were present. *M. bovis* did not grow in cell culture medium, since bacterial concentrations decreased after 6 h incubation in MEM-Earle medium (Figure 2A). L22/93 died after 54 h incubation in all experiments, while in the case of JF4278, a small quantity of bacteria could be detected in one case at 54 h (Figure 2A). Loss of *M. bovis* cells occurs during washing steps, therefore the inability of both strains to grow in MEM-Earle medium without washing steps was confirmed in a previous study (Bürgi et al., 2018). However, growth in spent MEM-Earle medium, i.e., medium pre-incubated with eukaryotic cells, cannot be ruled out as is seen for Bomac cells (Bürgi et al., 2018). Since *M. bovis* could only require the presence of eukaryotic metabolites to grow in MEM-Earle medium (Bürgi et al., 2018), “growth in co-culture” rather than “cell-associated growth” best describes the observed growth. When no gentamicin is added, both *M. bovis* strains survived in co-culture with bovine epithelial cell types, and a slight increase of mycoplasmal concentration was seen after 54 h of incubation compared to 0 and 6 h post-infection (Figure 2B). A significant difference in bacterial counts between JF4278 and L22/93 was detected with MDBK cells 6 h post-infection. Furthermore, 54 h post-infection, a significantly higher concentration of JF4278 compared to L22/93 was observed with MDBK and PECT cells. Additionally, higher concentrations of JF4278 were reached with MDBK cells compared to primary cells, while higher concentrations were observed in co-culture with bMec cells than with PECT cells (Figure 2B). The efficient killing of mycoplasmas with 3 h gentamicin treatment is shown in Figure 3A. After the gentamicin treatment, no viable bacteria could be recovered without co-cultivation with eukaryotic cells (Figure 3A). The gentamicin protection assay is shown in Figure 3B. When epithelial cells were present, a small amount of viable *M. bovis* was detected 6 h post-infection. Since gentamicin was shown to efficiently kill all “unprotected” mycoplasmas, the recovered bacteria 6 h post-infection correspond to the number of *M. bovis* that invaded bovine epithelial cells. No significant difference in the number of recovered bacteria was observed among the different cell types or *M. bovis* strains at this time point (Figure 3B). Regarding the invasion rates of the two *M. bovis* strains, they were comparable in all three epithelial cell types.

### Growth of *M. bovis* strain L22/93 is strongly reduced in co-culture with bovine mammary gland epithelial cells compared to other epithelial cell types

Growth of *M. bovis* in co-culture with bovine epithelial cells during the gentamicin protection assay was assessed 54 h post-infection. A significantly higher concentration of JF4278 was observed in co-culture with MDBK cells compared to PECT cells (Figure 3B). Additionally, significantly higher concentrations of JF4278 compared to L22/93 were measured in co-culture with all epithelial cell types (Figure 3B). This observation was even more pronounced with bMec cells (Figure 3B). Fifty-four hours post-infection, co-culture of L22/93 with bMec cells reached 300X and 60X lower titers in MDBK and PECT, respectively. On the other hand, differences in titers of JF4278 54 h post-infection were not >10-fold among epithelial cells.

### Confocal fluorescence microscopy reveals intra- and extracellular localization of *M. bovis*

**Figure 4:**
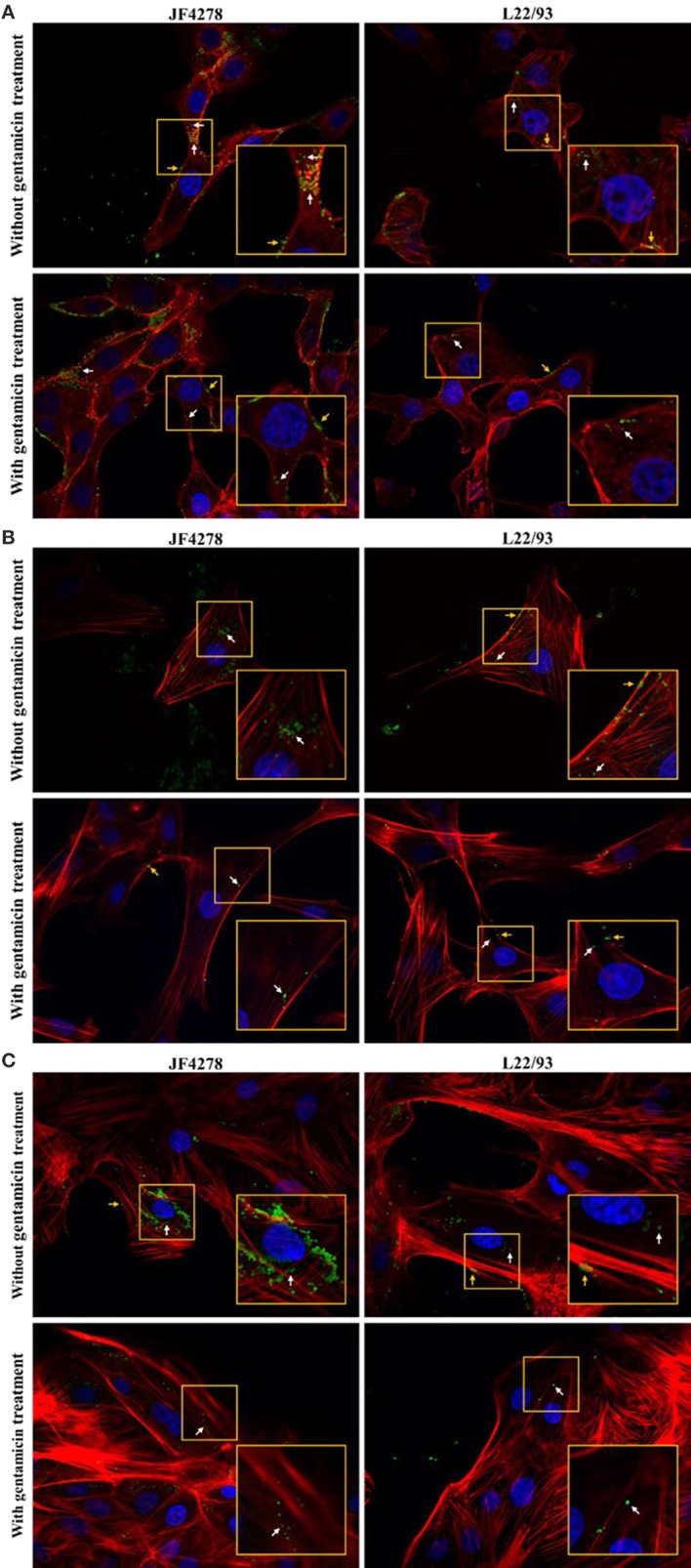
Confocal fluorescence microscopy of cell infections and gentamicin protection assays with fixed and permeabilized cells. Time point 54 h post-infection. MDBK cells **(A)**, PECT cells **(B)**, and bMec cells **(C)**. Stained nuclei are in blue, F-actin is in red, and mycoplasmas are in green. Images were merged and the magnification was 600X. The two upper images of each figure represent infected cells without gentamicin treatment. The two lower images represent infected cells with gentamicin treatment. The inset represents an area of interest and is shown in two-fold magnification in each image. Orange arrows indicate extracellular and cell-associated *M. bovis*. White arrows indicate intracellular *M. bovis*.

**Figure 5:**
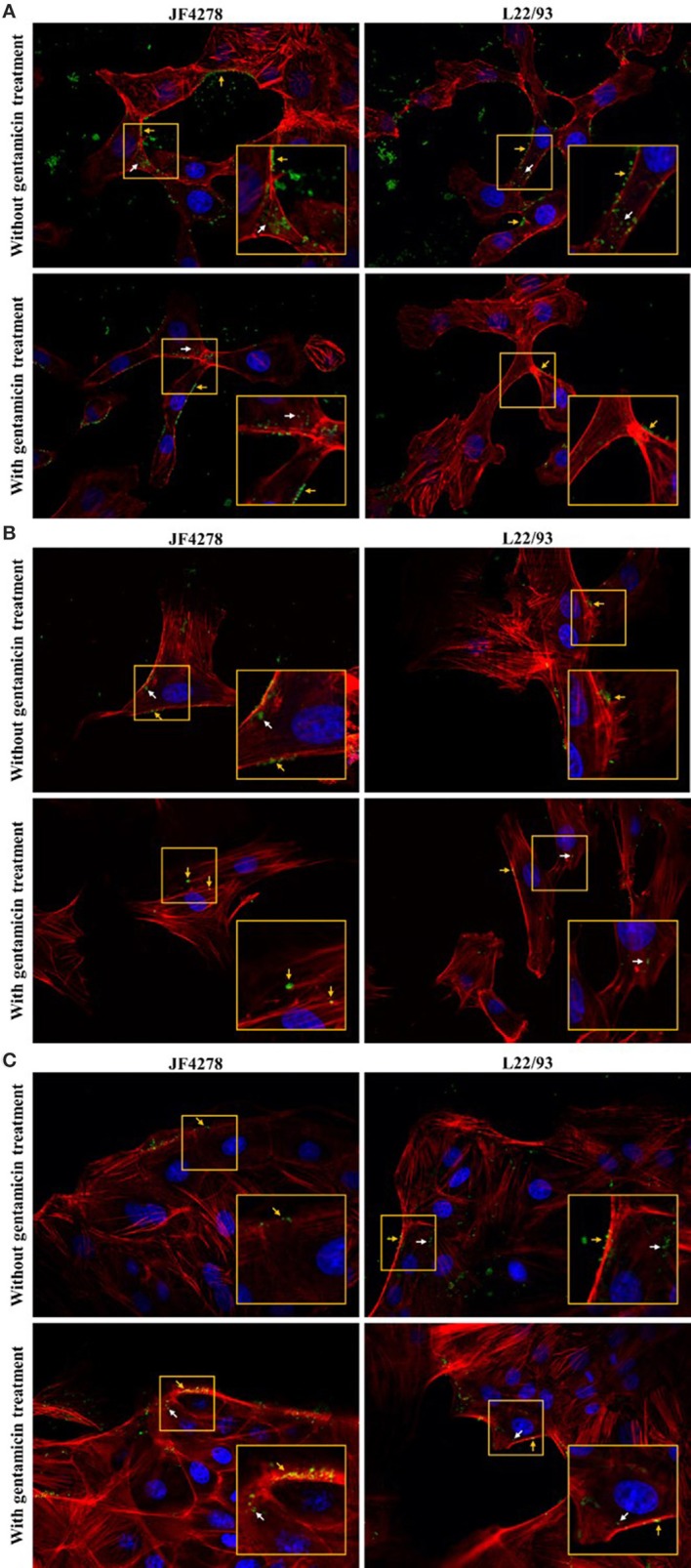
Confocal fluorescence microscopy of cell infections and gentamicin protection assays with fixed cells. Time point 54 h post-infection. MDBK cells **(A)**, PECT cells **(B)**, and bMec cells **(C)**. Stained nuclei are in blue, F-actin is in red, and mycoplasmas are in green. Images were merged and the magnification was 600X. The two upper images of each figure represent infected cells without gentamicin treatment. The two lower images represent infected cells with gentamicin treatment. The inset represents an area of interest and is shown in two-fold magnification in each image. Orange arrows indicate extracellular and cell-associated *M. bovis*. White arrows indicate intracellular *M. bovis*.

Confocal fluorescence microscopy was used to confirm extra- and intracellular localization of *M. bovis* after cell infection and gentamicin protection assays. Fifty-four hours post-infection, cells were either fixed or fixed and then permeabilized. Pictures from uninfected cells were acquired and no unspecific staining for *M. bovis* was observed (Supplementary Figure 2). Without gentamicin treatment, extracellular and cell-associated as well as intracellular bacteria were observed in all cell types (Figure 4). No obvious difference in the localization of bacteria between the two *M. bovis* strains was detected. In the gentamicin protection assays, extracellular and intracellular bacteria were visualized at 54 h post-infection (Figure 4). Moreover, in gentamicin-treated and unpermeabilized cells, *M. bovis* was observed outside of cells (Figure 5). Furthermore, some intracellularly localized bacteria could be detected in fixed cells, indicating some permeabilization of cells during the fixation step (Figure 5). The formaldehyde solution used in the fixation step contains 0.4% methanol as a stabilizer, hence a weak permeabilization of the cell membrane occurring during fixation cannot be ruled out. In general, more bacteria were detected when infection was performed with JF4278 than with L22/93 (Figures 4, 5). Additionally, more mycoplasmas were found to be present in samples initially not treated with gentamicin compared to samples from the gentamicin protection assay (Figures 4, 5). These differences between the two strains and treatments are in line with the CFUs/well values counted during cell infections (Figure 2B) and gentamicin protection assays (Figure 3B).

### *M. bovis* strain L22/93 has minor effects on bMec cells, but induces apoptosis or cytotoxic effects in other bovine epithelial cells

**Figure 6:**
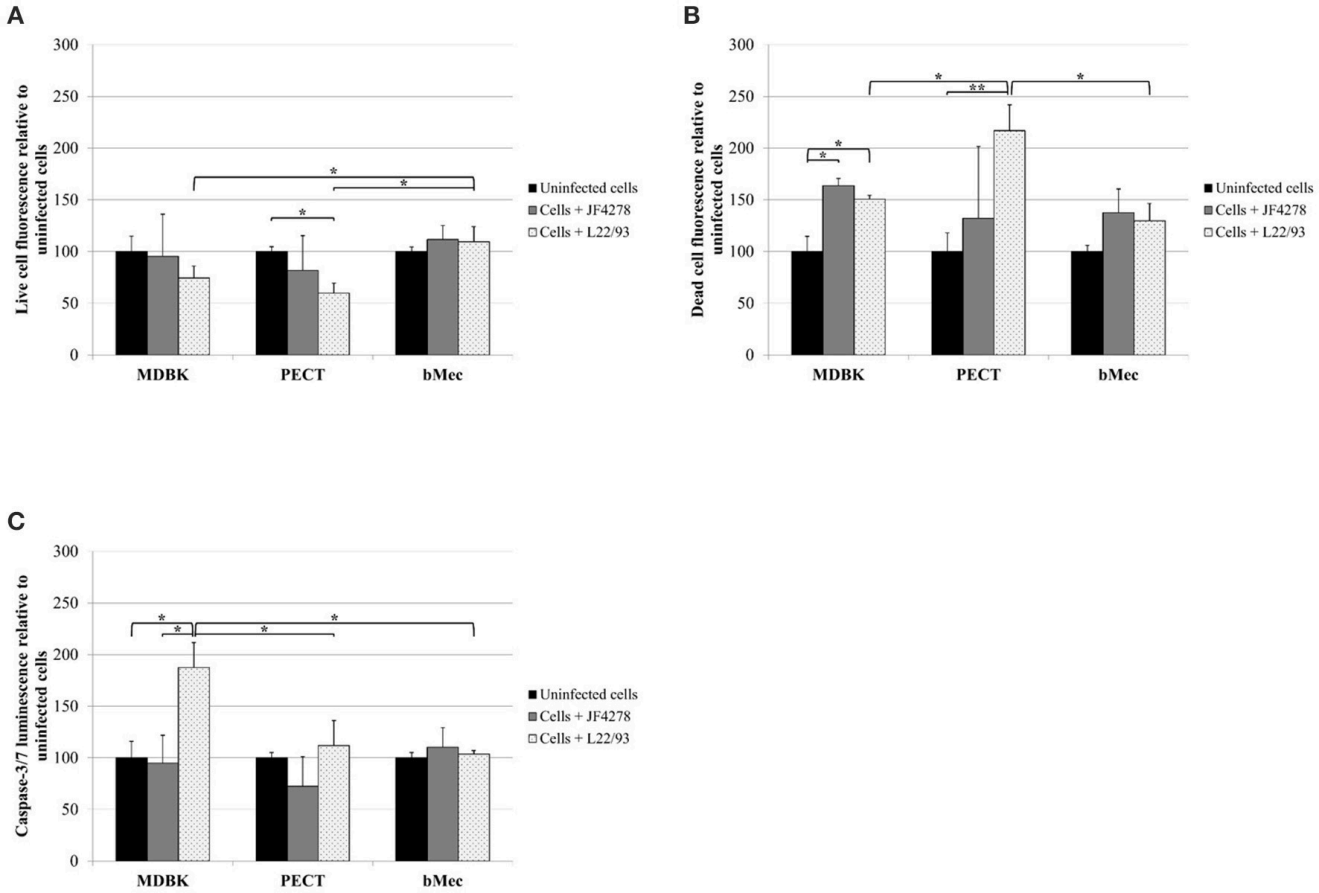
ApoTox-Glo™ Triplex assay. Viability **(A)**, cytotoxicity **(B)**, and apoptosis induction in eukaryotic cells **(C)**. Time point 24 h post-infection. Black columns correspond to uninfected cells, gray columns correspond to strain JF4278, while spotted columns correspond to strain L22/93. The x-axis indicates the different bovine epithelial cell types used. The y-axis represents the values for the respective test relative to uninfected cells. The values obtained for each cell type infected with *M. bovis* were normalized to values of the corresponding uninfected cells of each cell type. The data shown are the mean values of duplicates from three independent experiments. Standard deviations of measurements are indicated as vertical bars. Statistical analysis of the two strains within each cell type and the same strain between different cell types are shown. *P < 0.05, **P < 0.01.

Viability, cytotoxicity, and induction of apoptosis in bovine epithelial cells after infection with *M. bovis* were assessed using the ApoTox-Glo™ Triplex assay. Fluorescence and luminescence intensity values were expressed relative to the corresponding uninfected cells (Figure 6). Cell viability after infection with strain JF4278 did not drastically change in the tested cell types compared to uninfected cells (Figure 6A). However, viability of MDBK and PECT cells decreased after infection with strain L22/93 to 74 and 60% relative to uninfected cells (Figure 6A). As strain L22/93 did not reduce viability of bMec cells, a significant difference to the relative values for MDBK and PECT cells infected with L22/93 was detected (Figure 6A). Cytotoxicity was significantly increased after *M. bovis* infection in MDBK cells infected with both strains and in PECT cells with strain L22/93 (Figure 6B). L22/93 showed a higher cytotoxic effect in PECT cells compared with MDBK and bMec cells (Figure 6B). Efficiency of caspase-3/7 activation was assessed with staurosporine treatment of epithelial cells (Supplementary Figure 3). Apoptosis was significantly induced only in MDBK cells after infection with strain L22/93 (Figure 6C). Strain L22/93 strongly induced cytotoxicity in PECT cells and apoptosis in MDBK cells but not with bMec cells. This is in line with the reduced viability values in MDBK and PECT cells compared to the bMec cells after infection with L22/93.

### *M. bovis* infection has variable, dose-dependent effects on proliferation of primary epithelial cells

**Figure 7:**
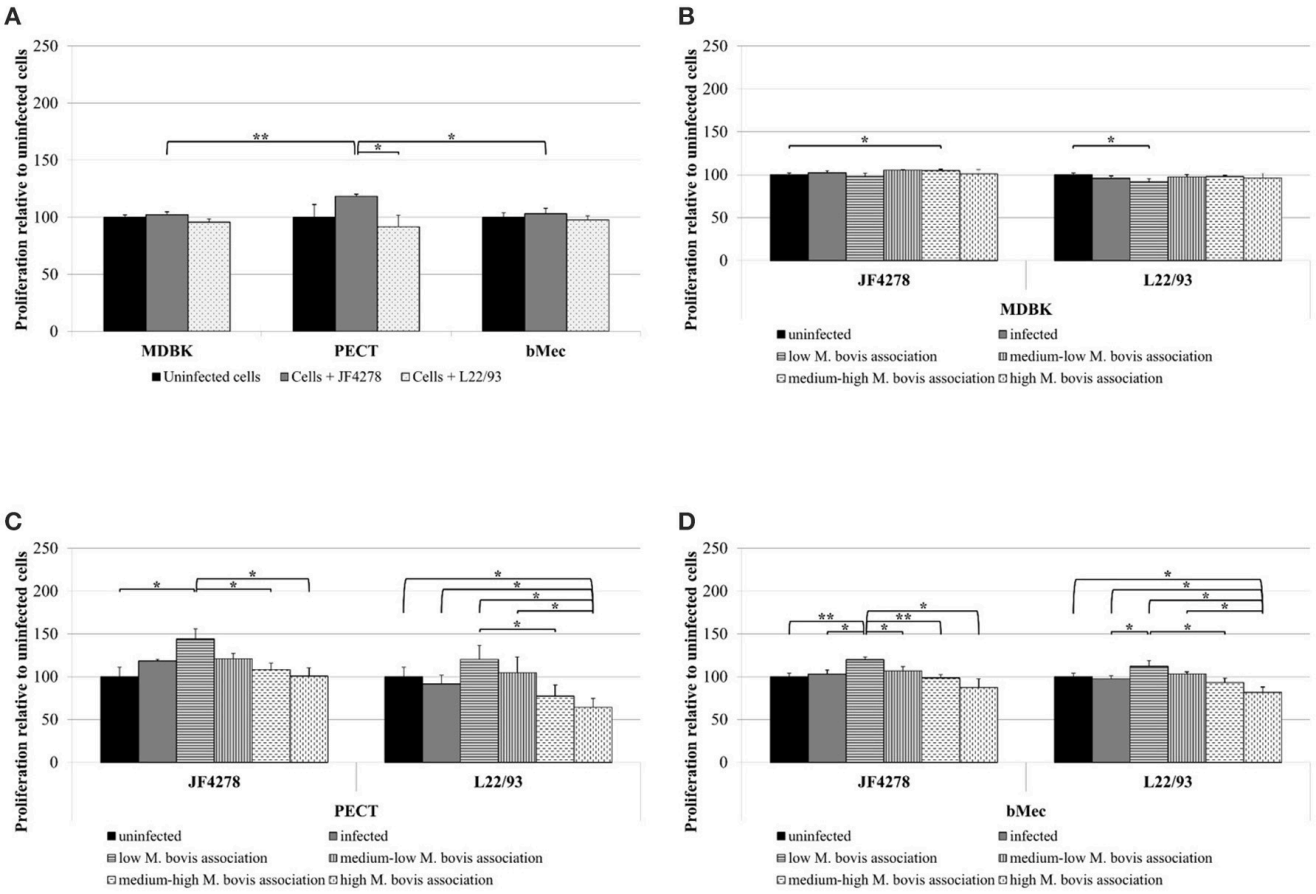
Proliferation assay. Total cell proliferation due to *M. bovis* **(A)**. Cell proliferation dependent on the amount of bacteria associated with each cell (MDBK: **B**, PECT: **C**, and bMec: **D**). Black columns correspond to uninfected cells, gray columns correspond to strain JF4278, while spotted columns correspond to strain L22/93 **(A)**. In panels **(B–D)**, gray columns correspond to *M. bovis* infection. Additionally, gray columns with horizontal or vertical lines and white columns with horizontally or vertically dashed lines correspond to low, medium-low, medium-high, and high *M. bovis* association, respectively. The x-axis indicates the different bovine epithelial cell types and the *M. bovis* strain. The y-axis represents the values for proliferation relative to uninfected cells. The data shown are the mean values of duplicates from three independent experiments. Standard deviations of measurements are indicated as vertical bars. In panel **(A)**, statistical analysis of the two strains within each cell type and the same strain between different cell types are shown. In Figures **(B–D)**, statistical analysis of proliferation values for individual cell types dependent on *M. bovis* association for the same strain are shown.*P < 0.05, **P < 0.01.

Proliferation of bovine cells after infection with *M. bovis* was measured using a high-throughput image analysis approach. For uninfected and infected cells, a total of minimum 25,818 (PECT cells infected with JF4278) and maximum 42,351 (bMec cells infected with L22/93) individual cells were analyzed. The percentage of proliferating cells was calculated and expressed relative to uninfected cells. To measure the amount of bacteria associated with each cell, a pseudo-cell was defined around each nucleus (example for MDBK cells in Supplementary Figure 1). Afterwards, the intensity of the fluorescent signal for *M. bovis* within this cell was quantified and cells were grouped in quartiles accordingly. In PECT cells, proliferation was increased after infection with strain JF4278 when compared to strain L22/93 (Figure 7A). Moreover, JF4278 induced more cell proliferation in PECT cells than in MDBK and bMec cells (Figure 7A). The results were then analyzed taking into account the amount of bacteria associated with each cell. While for primary cells dose dependent effects were observed, differences in MDBK cells were marginal (Figures 7B-D). JF4278 induced proliferation in primary cells compared to uninfected cells when small quantities of bacteria were associated with each cell (Figures 7C,D). This observation was not statistically significant for L22/93 (Figures 7C,D). For both *M. bovis* strains, a step-wise reduction in proliferating eukaryotic cells was observed in PECT and bMec cells associated with an increased amount of *M. bovis* (Figures 7C,D). Moreover, a significant reduction of proliferation was observed for primary cells with the highest L22/93 association compared to uninfected cells (Figures 7C,D).

## Discussion

Tissue predilection and cell permissivity of specific *M. bovis* strains are not known, and the cause for the increased severity of mastitis cases observed in Switzerland remains elusive (Bürki et al., 2016). Recently, the genetic characterization of *M. bovis* isolates from cattle and bison suggested host-specific genotypes (Register et al., 2015). It was later shown that *M. bovis* isolates from bison and cattle display differential effects on inhibition of cell proliferation and delay of apoptosis in bovine and bison blood cells (Suleman et al., 2016). Invasiveness of *M. bovis* isolates from bison was shown to be reduced compared to an isolate from a calf in the EBL cell line but not in the EBTr cell line (Suleman et al., 2016). This observation has not been explained and should be addressed by additional experiments. In this study, we investigated adhesion, cell invasion, cytotoxicity, induction of apoptosis, and cell proliferation in bovine epithelial cells using two *M. bovis* strains.

Both strains of *M. bovis* were able to adhere to all bovine epithelial cells. The adherence rate was in the range of previous studies (Sachse et al., 1996; Thomas et al., 2003a,b). However, our quantitation protocol has the advantage of not requiring specialized training or waste management as is necessary when experiments involve radioactive metabolic labeling. Both strains showed higher adherence to the cell line compared to primary cells (Figure 1) but JF4278 displayed higher adhesion rates than L22/93 to all three epithelial cells. These experiments did not reveal adhesion predilection of one strain to a specific cell type. A previous study was also unable to associate adherence tropism of certain cells types to *M. bovis* isolates from different organs.

Invasion of epithelial cells by *M. bovis* was assessed by two complementary methods, gentamicin protection assays and confocal fluorescence microscopy. Both assays led to the identification of intracellular bacteria (Figures 3B, 4). These findings support previous *in vitro* and *in vivo* observations with epithelial cells from the respiratory tract (Rodríguez et al., 1996; Maeda et al., 2003; Bürki et al., 2015b; Suleman et al., 2016). However, *M. bovis* was not detected in the cytosol of epithelial cells of the udder collected from pathological material (Stanarius et al., 1981; Radaelli et al., 2011), while invasion of bMec cells was observed (Figures 3B, 4C). The *in vivo* relevance of invasion of epithelial mammary gland cells has not yet been defined. These results suggest that the “severe mastitis phenotype” is not associated with differential invasiveness of the two tested *M. bovis* strains. However, after 54 h of co-infection with bMec cells in the gentamicin protection assay, the L22/93 strain has a significant lower titer compared to JF4278 (Figure 3B). This significant difference in titers between the two strains was found to be more prominent in co-culture with bMec cells compared to the other cell types. This cannot be explained by the reaching of a growth plateau. Indeed, at the same time point without supplementation of gentamicin, approximately 10^5^ L22/93 were recovered (Figure 2B). Moreover, no statistically relevant differences were observed in the cytotoxic effect of both strains toward bMec cells. Further studies will be necessary to assess if this discrepancy is a true variation in generation time between the two strains in co-culture with bMec cells. Confocal fluorescent microscopy showed *M. bovis* inside and outside bovine cells (Figure 4). In a previous study using a similar protocol, no extracellular mycoplasmas were detected after the initial treatment of cells with gentamicin (Bürki et al., 2015b). The discrepancy observed between the two studies might be explained by technical reasons. In the present study a rabbit polyclonal antiserum directed against PG45^T^ was used as primary antibody, while in the previous study a mouse monoclonal IgG1 primary antibody was used (Bürki et al., 2015b). The polyclonal serum might be more sensitive than monoclonal antibodies since it targets more epitopes. Additionally, the previous experiments were performed using epifluorescence and not confocal microscopy. It is not clear whether the mycoplasmas detected outside cells after the gentamicin treatment evaded cells and reattached, as suggested with the closely related *M. agalactiae*, or if they derive from lysed eukaryotic cells (Hegde et al., 2014). After 24 h of infection with *M. bovis*, cytotoxicity was slightly increased in all the cell types (Figure 6B). Therefore, cell disruption cannot be ruled out as a source of extracellular mycoplasmas after 54 h of infection. Collectively, the confocal fluorescence microscopy shows occasional intracellular localization of *M. bovis* in epithelial cell types and confirms the results from the gentamicin protection assay.

Strain L22/93 showed a higher cytotoxic effect toward PECT cells and induced more apoptosis in MDBK cells compared to bMec cells (Figure 6). Although this is not definitive evidence, it might be linked to the reduced growth of L22/93 in co-culture with bMec cells (Figure 3B). Indeed, lower mycoplasma counts during infection could lead to decreased severity of the disease (Nilsson et al., 2010). In *in vivo* experiments, low infectious doses of *M. bovis* produced significantly fewer lung lesions and cytopathic effects compared to high infectious doses (Prysliak et al., 2011). Similarly, another *in vitro* study showed that high *M. bovis* counts reduced viability and increased expression of pro-inflammatory cytokines in PBMCs (Gondaira et al., 2015). Additionally, an *M. bovis* encoded secretory nuclease was shown to induce cytotoxicity and apoptosis in a dose dependent manner in Bomac cells (Zhang et al., 2016). The growth rate of specific strains of *M. bovis* in organs or cells might be a relevant parameter in the development of lesions. However, JF4278 did not significantly alter viability of infected cells (Figure 6A). Since JF4278 generally reaches higher titers than strain L22/93 in co-culture with epithelial cells (Figure 3B), a sole dependency on the amount of mycoplasmas on the reduction of cell viability is questionable. Additionally, bMec cells were found to be contaminated with BVDV. Although interactions with BVDV cannot be totally excluded, a previous study showed that co-infection with BVDV in Bomac cells did not substantially change cell invasion, mycoplasmal growth or cytotoxicity after infection with *M. bovis* (Bürgi et al., 2018). Furthermore, cytotoxicity was slightly but often not significantly increased after infection with *M. bovis* in all cell types (Figure 6B). As mentioned above, apoptosis was induced in MDBK cells infected with L22/93. However, induction of apoptosis was found to be unchanged in primary epithelial cells infected with either strain of *M. bovis* (Figure 6C). Therefore, other cell death routes might be of importance for *M. bovis* infected epithelial cells. Bacterial infections were shown to trigger caspase-1 associated pyroptosis and TNF-induced necrosis (Blériot and Lecuit, 2016). Additionally, activated caspase-1 is involved in the cleavage and activation of pro-IL1-β and pro-IL-18 (Blériot and Lecuit, 2016). Since, epithelial cells were shown to be the source of pro-inflammatory cytokines like TNF-α and IL1-β after infection with *M. bovis* (Zbinden et al., 2015; Wang et al., 2016; Gondaira et al., 2018), these cell death routes could be of importance for infected epithelial cells.

Cytotoxicity due to *M. bovis* was not as evident as with *M. mycoides mycoides* (Pilo et al., 2005). Cell proliferation was tested to assess if cell death might be concealed/obscured by increased cell division. Cell proliferation was not increased by *M. bovis* compared to uninfected cells. Therefore, the cytotoxicity measured was not associated with cell proliferation. However, low concentrations of JF4278 increased proliferation of primary epithelial cells significantly compared to uninfected cells (Figures 7C,D), but when cells are associated with large amounts of mycoplasmas the proliferative effect is lost. High amounts of strain L22/93 associated with cells even led to an anti-proliferative response of primary epithelial cells. The same observation was also previously made with PBMCs (Suleman et al., 2018). However, the *in vivo* relevance of this finding is not clear.

In summary, this study showed no adherence predilection to specific epithelial cells associated with strains JF4278 and L22/93. *M. bovis* is able to invade different epithelial cells *in vitro*, including epithelial mammary gland cells. However, no differences in invasion rates were observed between the two strains. Furthermore, a dose dependent effect of *M. bovis* on proliferation of primary epithelial cells was observed. Strain L22/93 showed less severe cytopathic effects on bMec cells compared to MDBK and PECT cells and this could be linked to the bacterial titers measured in co-culture with the respective epithelial cells. However, a direct link between bacterial titers reached during gentamicin protection assays and the reduced viability of PECT and MDBK cells would require further proof. Future studies should focus on the induction of cell death pathways in different infected cell types. Moreover, two recently published genomics studies characterized several factors potentially related to *M. bovis* virulence (Parker et al., 2016; Rasheed et al., 2017). Functional genomic studies will be required in the future.

## Data availability statement

The raw data supporting the conclusions of this manuscript will be made available by the authors without undue reservation to any qualified researcher.

## Author contributions

CJ, SB, AS, OW, MS, and PP designed the experiments. CJ, AS, and SB performed the experiments. CJ, SB, AS, and PP analyzed the data. CJ drafted the manuscript. All authors helped in writing the manuscript and critically revised it. All authors read and approved the final manuscript.

### Conflict of interest statement

The authors declare that the research was conducted in the absence of any commercial or financial relationships that could be construed as a potential conflict of interest.

## Supplementary material

Supplementary Figure 1 Example of image segmentation for the proliferation assay. Images of uninfected and *M. bovis* infected MDBK cells. Time point 24 h post-infection. DAPI (Hoechst stained nuclei), FITC (Alexa Fluor® 488 stained *M. bovis*), Cy5 (Andy Fluor 647 stained DNA with EdU), and bright-field images (morphology of cells) are shown. The magnification was 200X. Images were analyzed using the INCell Investigator 1.6.2 software (GE Healthcare). After image segmentation on the DAPI signal (blue circle around the nuclei), a pseudo-cell was defined around the nucleus by expanding the nuclear mask by 44.4 μm in diameter (yellow pseudo-cell circle for each nucleus). To ensure that adjacent cells were separated, a clump-breaking algorithm was applied (no overlapping pseudo-cells). Within the area of each of the defined pseudo-cells the intensity of the Cy5 and FITC signal was measured (not shown in this picture; values were exported to a MS Excel file). In an additional step, Cy5 positive cells were checked for overlapping DAPI and Cy5 signals (red circle around Cy5 positive nuclei). All experiments were performed three times in duplicates. In the case of the uninfected MDBK cells a total of 29,842 individual cells were analyzed, whereas for MDBK cells infected with strain JF4278 a total of 30,762 cells were analyzed.

Supplementary Figure 2 Confocal fluorescence microscopy of cell infections and gentamicin protection assays with uninfected cells. Time point 54 h post-infection. MDBK cells **(A)**, PECT cells **(B)**, and bMec cells **(C)**. Stained nuclei are in blue, F-actin is in red, and mycoplasmas are in green. Images were merged and the magnification was 600X. The two upper images of each figure represent uninfected cells without gentamicin treatment. The two lower images of each figure represent uninfected cells with gentamicin treatment. Fixed cells are shown in the left images, whereas fixed and permeabilized cells are shown in the images on the right.

Supplementary Figure 3 Apoptosis induction with staurosporine. Apoptosis induction in uninfected cells by staurosporine. Black columns correspond to uninfected cells, checkered columns correspond to uninfected cells + staurosporine. The x-axis indicates the different bovine epithelial cell types used. The y-axis represents the values for the apoptosis induction test relative to uninfected cells. The values obtained for each cell type treated with staurosporine were normalized to values of the corresponding untreated cells of each cell type. The data shown are the mean values of duplicates from three independent experiments. Standard deviations of measurements are indicated as vertical bars. Statistical analysis between untreated and staurosporine-treated cells within each cell type are shown. ^*^*P* < 0.05, ^**^*P* < 0.01.

Supplementary Figure 4 Efficient killing of *M. bovis* by gentamicin. Survival of *M. bovis* in MEM-Earle medium with gentamicin treatment without cells. Gray columns correspond to strain JF4278, while spotted columns correspond to strain L22/93. The x-axis indicates the different time points and the gentamicin concentration used. The y-axis represents the log10 CFU/well of *M. bovis*. The data shown are the mean values of duplicates. Standard deviations of measurements are indicated as vertical bars.

Supplementary Table 1 Overview of the infection parameters for the different assays.

Supplementary Table 2 Real-time qPCR results of uninfected bovine epithelial cells.
