# Supplementary figures and images for: Bovine Epithelial in vitro Infection Models for Mycoplasma bovis
Source: Front Cell Infect Microbiol. 2018 Sep 18;8:329. doi: 10.3389/fcimb.2018.00329 (PMC6153342; doi:10.3389/fcimb.2018.00329)

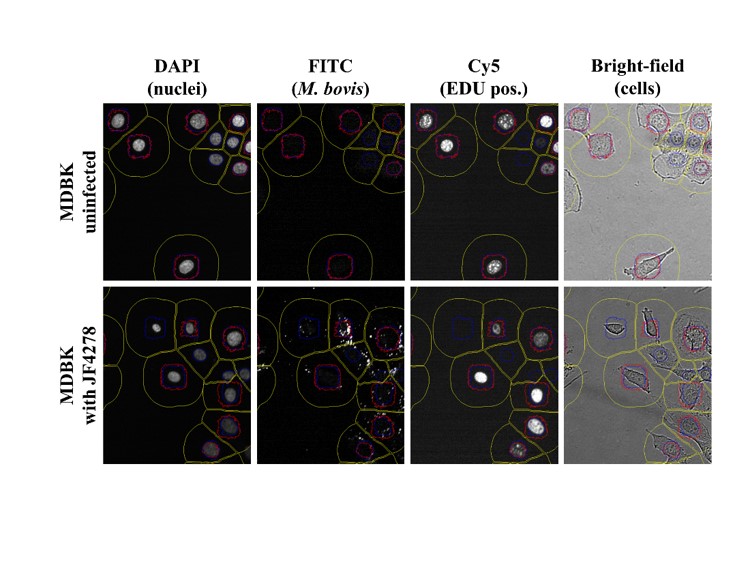

Supplement: Supplementary Figure 1 — Example of image segmentation for the proliferation assay. Images of uninfected and M. bovis infected MDBK cells. Time point 24 h post-infection. DAPI (Hoechst stained nuclei), FITC (Alexa Fluor® 488 stained M. bovis), Cy5 (Andy Fluor 647 stained DNA with EdU), and bright-field images (morphology of cells) are shown. The magnification was 200X. Images were analyzed using the INCell Investigator 1.6.2 software (GE Healthcare). After image segmentation on the DAPI signal (blue circle around the nuclei), a pseudo-cell was defined around the nucleus by expanding the nuclear mask by 44.4 μm in diameter (yellow pseudo-cell circle for each nucleus). To ensure that adjacent cells were separated, a clump-breaking algorithm was applied (no overlapping pseudo-cells). Within the area of each of the defined pseudo-cells the intensity of the Cy5 and FITC signal was measured (not shown in this picture; values were exported to a MS Excel file). In an additional step, Cy5 positive cells were checked for overlapping DAPI and Cy5 signals (red circle around Cy5 positive nuclei). All experiments were performed three times in duplicates. In the case of the uninfected MDBK cells a total of 29,842 individual cells were analyzed, whereas for MDBK cells infected with strain JF4278 a total of 30,762 cells were analyzed. [file Image_1.JPEG]

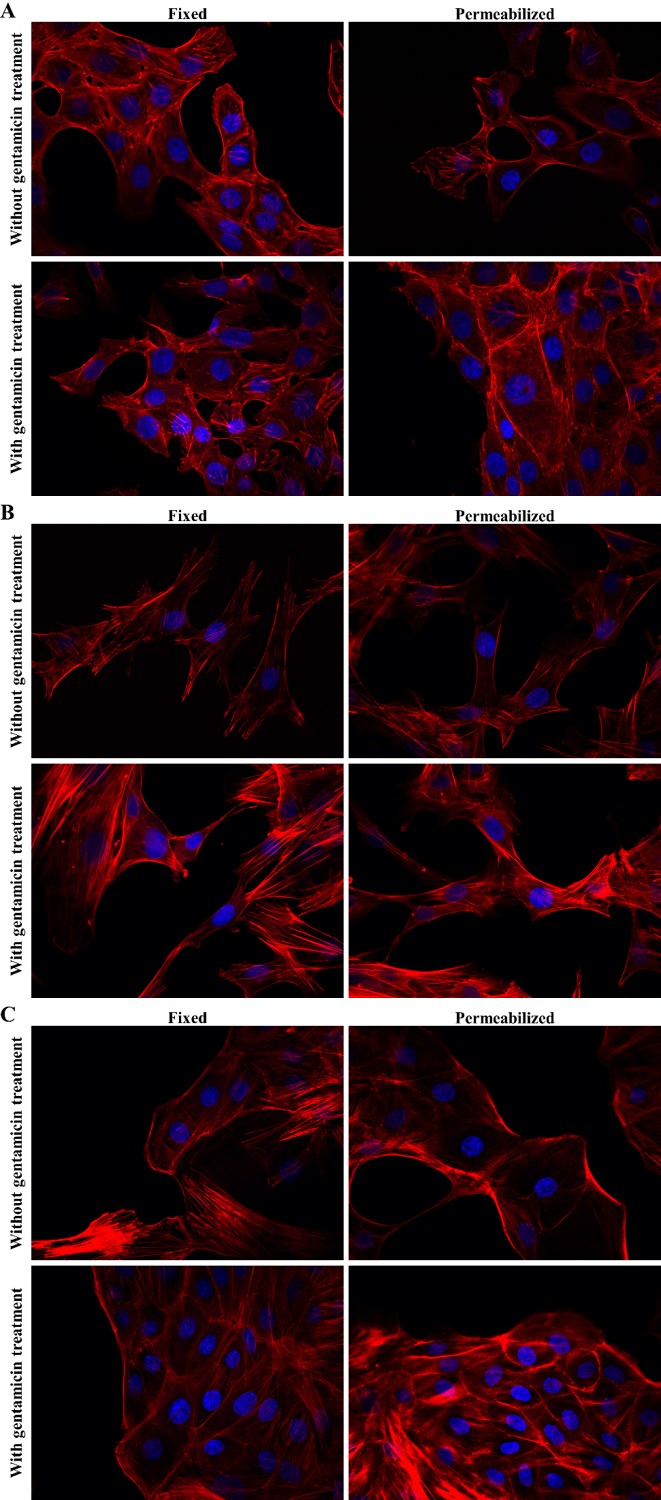

Supplement: Supplementary Figure 2 — Confocal fluorescence microscopy of cell infections and gentamicin protection assays with uninfected cells. Time point 54 h post-infection. MDBK cells (A), PECT cells (B), and bMec cells (C). Stained nuclei are in blue, F-actin is in red, and mycoplasmas are in green. Images were merged and the magnification was 600X. The two upper images of each figure represent uninfected cells without gentamicin treatment. The two lower images of each figure represent uninfected cells with gentamicin treatment. Fixed cells are shown in the left images, whereas fixed and permeabilized cells are shown in the images on the right. [file Image_2.JPEG]

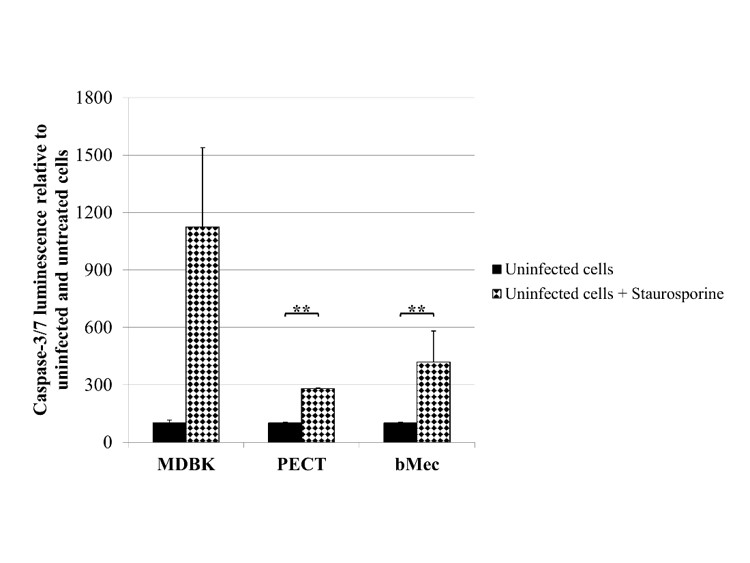

Supplement: Supplementary Figure 3 — Apoptosis induction with staurosporine. Apoptosis induction in uninfected cells by staurosporine. Black columns correspond to uninfected cells, checkered columns correspond to uninfected cells + staurosporine. The x-axis indicates the different bovine epithelial cell types used. The y-axis represents the values for the apoptosis induction test relative to uninfected cells. The values obtained for each cell type treated with staurosporine were normalized to values of the corresponding untreated cells of each cell type. The data shown are the mean values of duplicates from three independent experiments. Standard deviations of measurements are indicated as vertical bars. Statistical analysis between untreated and staurosporine-treated cells within each cell type are shown. *P < 0.05, **P < 0.01. [file Image_3.JPEG]

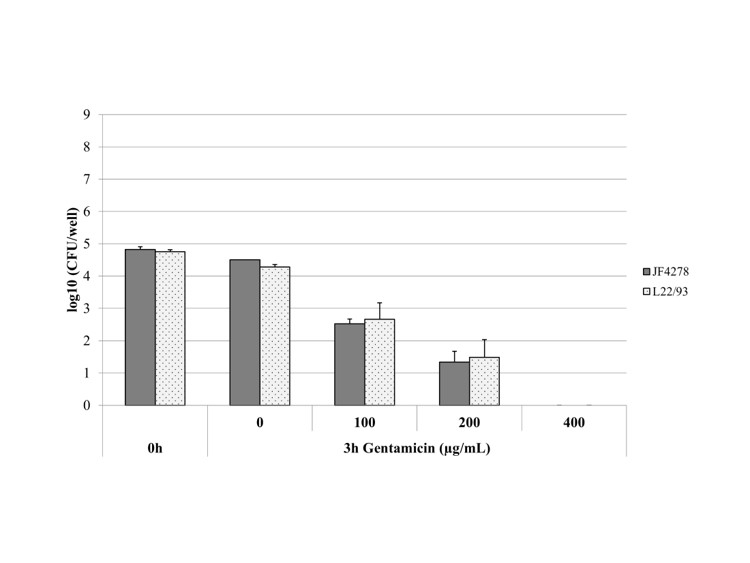

Supplement: Supplementary Figure 4 — Efficient killing of M. bovis by gentamicin. Survival of M. bovis in MEM-Earle medium with gentamicin treatment without cells. Gray columns correspond to strain JF4278, while spotted columns correspond to strain L22/93. The x-axis indicates the different time points and the gentamicin concentration used. The y-axis represents the log10 CFU/well of M. bovis. The data shown are the mean values of duplicates. Standard deviations of measurements are indicated as vertical bars. [file Image_4.JPEG]
