# Supplementary material for: Bovine Epithelial in vitro Infection Models for Mycoplasma bovis
Source: Front Cell Infect Microbiol. 2018 Sep 18;8:329. doi: 10.3389/fcimb.2018.00329 (PMC6153342; doi:10.3389/fcimb.2018.00329)
Supplement: Supplementary Table 1 — Overview of the infection parameters for the different assays. [file Table_1.docx]

Supplementary Table 1

Bovine Epithelial *in vitro* Infection Models for *Mycoplasma bovis*

Christoph Josi, Sibylle Bürki, Ana Stojiljkovic, Olga Wellnitz, Michael H. Stoffel, Paola Pilo^*^

*** Correspondence:** Paola Pilo: [paola.pilo@vetsuisse.unibe.ch](mailto:paola.pilo@vetsuisse.unibe.ch)

| **Experiment** | **Adhesion assay** | **Gentamicin protection assay** | **Fluorescence microscopy for *M. bovis*** | **ApoTox-Glo^TM^ Triplex assay** | **Proliferation assay** |
| --- | --- | --- | --- | --- | --- |
| **Plate** | 24-well^1^ | 24-well^1^ | 24-well^1^  with glass slides^2^ | 96-well^3^ | 96-well^3^ |
| **Cells**  **seeded / plate** | ̴ 2 x 10^6^ (MDBK)  ̴ 3 x 10^6^ (PECT & bMec) | ̴ 1 x 10^6^ (MDBK)  ̴ 1.5 x 10^6^ (PECT & bMec) | ̴ 0.3 x 10^6^ (MDBK & PECT)  ̴ 1.5 x 10^6^ (bMec) | ̴ 0.8 x 10^6^ (MDBK)  ̴ 1.2 x 10^6^ (PECT & bMec) | ̴ 0.3 x 10^6^ (MDBK)  ̴ 0.6 x 10^6^ (PECT & bMec) |
| **Counted cells at T = 0h / well** | ̴ 8 x 10^4^ | ̴ 4 x 10^4^ | ̴ 1 x 10^4^ (MDBK & PECT)  ̴ 3 x 10^4^ (bMec) | ̴ 7.5 x 10^3^ | ̴ 4 x 10^3^ |
| **MOI^4^** | 5.4 | 9.0 | 5.8 | 7.9 | 1.3 |
| **Infection time** | 30 minutes / 2 hours | 0 – 54 hours | 54 hours | 24 hours | 24 hours |

**Supplementary Table 1. Overview of the infection parameters for the different assays.** The Madin-Darby Bovine Kidney cell line (MDBK) and the two primary epithelial cell types, Primary Embryonic Calf Turbinate (PECT) cells and bovine mammary gland epithelial cells (bMec), were used for the experiments. *M. bovis* strains L22/93 and JF4278 were used to perform the experiments.

^1^ 24-well plates (TPP®, Trasadingen, Switzerland)

^2^ Round glass slides of 12 millimeter diameter and #1.5 thickness (Neuvitro, Vancouver, USA)

^3^ 96-well plates, µClear®, black chimney well with a clear bottom (Greiner Bio-One, Frickenhausen, Germany)

^4^ MOI (mulitplicity of infection) is defined as the number of added bacteria per individual host cell. The mean MOI of each assay is indicated.
