# Supplementary material for: Bovine Epithelial in vitro Infection Models for Mycoplasma bovis
Source: Front Cell Infect Microbiol. 2018 Sep 18;8:329. doi: 10.3389/fcimb.2018.00329 (PMC6153342; doi:10.3389/fcimb.2018.00329)
Supplement: Supplementary Table 2 — Real-time qPCR results of uninfected bovine epithelial cells. [file Table_2.docx]

Supplementary Table 2

Bovine Epithelial *in vitro* Infection Models for *Mycoplasma bovis*

Christoph Josi, Sibylle Bürki, Ana Stojiljkovic, Olga Wellnitz, Michael H. Stoffel, Paola Pilo^*^

*** Correspondence:** Paola Pilo: [paola.pilo@vetsuisse.unibe.ch](mailto:paola.pilo@vetsuisse.unibe.ch)

| **Real-time qPCR** | **C_T_ *M. bovis*** | **C_T_ 18S rRNA** |
| --- | --- | --- |
| **MDBK** | Undetermined | 22.08 (+/- 0.23) |
| **PECT** | Undetermined | 21.16 (+/- 0-14) |
| **bMec** | Undetermined | 18.29 (+/- 0.10) |
| **JF4278** | 24.46 (+/- 0.01) | Undetermined |
| **H_2_O** | Undetermined | Undetermined |

**Supplementary Table 2. Real-time qPCR results of uninfected bovine epithelial cells.** Uninfected epithelial cells were lysed as described for the adhesion assay. C_t_ values of *M. bovis* and 18S rRNA qPCR are shown from three experiments measured in duplicates. As a positive control for the *M. bovis* qPCR, a lysate of strain JF4278 was included.
